# Dysregulated H3K27 Acetylation Is Implicated in Fatty Liver Hemorrhagic Syndrome in Chickens

**DOI:** 10.3389/fgene.2020.574167

**Published:** 2021-01-11

**Authors:** Yaling Zhu, Qingjie Zeng, Fang Li, Haoshu Fang, Zhimin Zhou, Tao Jiang, Chao Yin, Qing Wei, Yujie Wang, Jiming Ruan, Jianzhen Huang

**Affiliations:** ^1^College of Animal Science and Technology, Jiangxi Agricultural University, Nanchang, China; ^2^Department of Pathophysiology, Anhui Medical University, Hefei, China; ^3^Laboratory Animal Research Center, College of Basic Medical Science, Anhui Medical University, Hefei, China

**Keywords:** fatty liver hemorrhagic syndrome, ChIP-seq, RNA-seq, H3K27ac, chicken

## Abstract

Epigenetic regulation of gene expression has been reported in the pathogenesis of metabolic disorders such as diabetes and liver steatosis in humans. However, the molecular mechanisms of fatty liver hemorrhagic syndrome (FLHS) in chickens have been rarely studied. H3K27ac chromatin immunoprecipitation coupled with high-throughput sequencing and high-throughput RNA sequencing was performed to compare genome-wide H3K27ac profiles and transcriptomes of liver tissue between healthy and FLHS chickens. In total, 1,321 differential H3K27ac regions and 443 differentially expressed genes were identified (| log2Fold change| ≥ 1 and *P*-value ≤ 0.05) between the two groups. Binding motifs for transcription factors involved in immune processes and metabolic homeostasis were enriched among those differential H3K27ac regions. Differential H3K27ac peaks were associated with multiple known FLHS risk genes, involved in lipid and energy metabolism (*PCK1*, *APOA1*, *ANGPTL4*, and *FABP1*) and the immune system (*FGF7*, *PDGFRA*, and *KIT*). Previous studies and our current results suggested that the high-energy, low-protein (HELP) diet might have an impact on histone modification and chromatin structure, leading to the dysregulation of candidate genes and the peroxisome proliferator-activated receptor (PPAR) signaling pathway, which causes excessive accumulation of fat in the liver tissue and induces the development of FLHS. These findings highlight that epigenetic modifications contribute to the regulation of gene expression and play a central regulatory role in FLHS. The PPAR signaling pathway and other genes implicated in FLHS are of great importance for the development of novel and specific therapies for FLHS-susceptible commercial laying hens.

## Introduction

Fatty liver hemorrhagic syndrome (FLHS) is a lipid metabolism disorder, which is characterized by a dramatic drop in egg production and increased mortality of commercial laying hens, in turn causing considerable economic losses (Hansen and Walzem, 1993; Trott et al., 2014; Rozenboim et al., 2016). Several factors have been reported to contribute to the development of FLHS, including heredity, environment, nutrition, toxic substances, and hormones (Schuman et al., 2000; Li et al., 2013). Among these, nutrition is considered the main cause of FLHS in the modern poultry industry, and 97% of affected chickens have large fat depots (Trott et al., 2014). Despite of the progress made in understanding the risk factors that contribute to FLHS, the epigenetic mechanism via which nutrition drives FLHS susceptibility remain elusive, and adaptive changes in epigenetic and transcriptional regulation play an important role in the phenotypic adaptation of cells to the environment (Corradin and Scacheri, 2014; Lee et al., 2014; Nammo et al., 2018).

Many reports support the notion that epigenomic dysregulation may influence transcriptional output and signaling pathways, providing a mechanistic basis for investigating its involvement in various common diseases, such as non-alcoholic fatty liver (NAFLD), diabetes, Alzheimer’s diseases, and others (Ling and Groop, 2009; Lee et al., 2014; Marzi et al., 2018; Nammo et al., 2018). For example, Lee et al. (2014) found that epigenetic manipulation through the metabolic pathway of one-carbon metabolism slows the progression of NAFLD. Ling and Groop (2009) suggested that genome-wide technologies for studying the gene expression and genetic variations in patients with type 2 diabetes have revealed a variety of new diabetes-related genes. Marzi et al. (2018) reported that differentially acetylated peaks were enriched for disease-related pathways and associated genes, a change which was involved in the development of amyloid-β and tau pathology in sporadic late-onset AD. To date, however, no systematic study has examined the regulatory modifications of FLHS in chicken.

H3K27ac, an epigenetic marker of active enhancers and promoters, is strongly associated with transcription factor binding and gene expression. Genome-wide H3K27ac profiles provide valuable information not only for annotating variants but also for understanding disease in both humans and animals (Creyghton et al., 2010; Marzi et al., 2018). Herein, we carried out chromatin immunoprecipitation combined with high-throughput sequencing (ChIP-Seq) for the H3K27ac marker and RNA sequencing to analyze the genome-wide H3K27ac profiles and liver transcriptomes of high-energy, low-protein (HELP) diet-induced FLHS chicken models and healthy birds with the aim of identifying transcription factor binding motifs, candidate genes, and pathways functionally related to FLHS.

## Materials and Methods

### Ethics and Consent

All the animals are raised in compliance with the care and use guidelines of experimental animals established by the Ministry of Agriculture of China. This study was approved by the Ethics Committee of Jiangxi Agricultural University and Anhui Medical University.

### Experimental Animals and Tissue Collection

To study the transcriptional programs and epigenomes of liver tissues from healthy and FLHS chickens, we first chose 90 healthy 155-day-old Hy-Line Brown layers with an average body weight of 1.5 kg. After 7 days of accommodation at temperature of 28°C, layers were randomly assigned as experimental and control groups, so that each group had 45 layers divided between three replicates (15 layers per replicate). Layers in the control group were fed a standard diet (Baker and Han, 1994), and those of the experimental group were fed a HELP diet (Supplementary Table 1). Then, we randomly dissected livers from three experimental and three control chickens at about 8 weeks of age, following the standardized sample collection protocols of the FAANG Project^1^ for following RNA-seq and ChIP-seq.

Phenotypic values are presented as mean ± standard deviation (*M* ± SD). Statistical comparisons of phenotypic values between the experimental and control groups were carried out using the Student’s *t*-test. Statistical differences were considered significant at *P* ≤ 0.05 and highly significant at *P* ≤ 0.01.

### Histopathological Examination

We dissected fresh liver samples and fixed them in 10% neutral buffered formalin. Samples were then routinely embedded in paraffin and stained with hematoxylin and eosin (H&E) as previously reported (Cinti, 2001). We used an optical microscope to observe stained sections and Image-Pro Plus 6.0 software to calculate the diameters of stained adipocytes.

### RNA Sequencing and Analysis of Differential Gene Expression

Total RNA was extracted from liver tissue using the TRIzol reagent (Invitrogen, United States). Extracted RNA was then used to construct cDNA libraries using the NEBNext^®^ Ultra^TM^ Directional RNA Library Prep Kit for Illumina^®^ (NEB, United States). On a HiSeq 4000 platform (Illumina) by Novogene (United States), 150-bp reads (paired-end) were generated at a depth of approximately 46.2 million reads (Supplementary Table 2).

We mapped filtered reads to the chicken reference genome Gallus_gallus-6.0 (Ensembl) using STAR-2.5.3a (Dobin et al., 2013). The featureCounts software (Liao et al., 2014) was used with “gene” as the feature and in strand-unaware mode. Lowly expressed genes (when the counts across 90% samples are lower than 2) were then removed, since those are usually more vulnerable to measurement errors as reported in our previous study (Zhu et al., 2020). The fragments per kilobase of transcript sequence per millions base pairs (FPKM) algorithm was used to normalize the expression of each gene. Differential expression analysis was performed using the DESeq2 R package (Love et al., 2014), and the resulting *P*-value was adjusted using Benjamini and Hochberg’s approach for controlling false discovery rates (Zhu et al., 2020). Bioinformatics analyses were performed in R version 3.5.1^2^.

### Differential ChIP-Seq Analysis of H3K27-Acetylated Regions

Chromatin immunoprecipitation samples, from the same samples used in RNA-seq, were prepared using the SimpleChIP^®^ Plus Enzymatic Chromatin IP Kit (Magnetic Beads, 9005) with 500 μg chromatin and 5 μg anti-H3K27ac antibody (Active motif, 39133), following protocols from https://www.encodeproject.org/about/experiment-guidelines/ and https://www.animalgenome.org/community/FAANG. The dissected tissues are treated with 37% formaldehyde to cross-link proteins covalently to DNA. This is followed by cell disruption and sonication to shear the chromatin to a target size of 100–300 bp (Nord et al., 2013). After protease and RNAse treatment, DNA was purified, and real-time quantitative polymerase chain reaction (RT-PCR) was performed. Chromatin immunoprecipitation (ChIP) and input library construction and sequencing procedures were carried out according to Illumina protocols with minor modifications (Illumina, San Diego, CA, United States) to obtain approximately 35.5 million reads per library (Supplementary Table 2).

Trimmed clean reads were mapped to the chicken reference genome Gallus_gallus-6.0 (Ensembl) using the Burrows–Wheeler Aligner (BWA) (Abuin et al., 2015), allowing two mismatches. After that, a Model-based Analysis for ChIP-Seq version 2.1.0 (MACS 2.1.0) peak caller was used to determine H3K27ac-enriched regions by setting the *q*-value threshold as 1e-5 (Zhang et al., 2008). Peak files were then sorted and analyzed for intersections using bedtools version 2.27.0 (Quinlan and Hall, 2010). Peak regions were intersected for all peaks across data sets, with a distance of less than 1 kb between summits (defined as the base position with the highest coverage within the peak region) used as the merge criteria, following similar steps set by a previous report (Nord et al., 2013). Table 1 shows a summary of statistics for all ChIP-seq data sets.

**TABLE 1:** Effect of fatty liver hemorrhagic syndrome on liver index, hepatic triglyceride, and hepatic total cholesterol in chickens^1^.

Parameters^2^	Control	Experimental
Liver index (‰)	14.12 ± 0.70^*A*^	20.03 ± 0.68^*B*^
Hepatic TG (mmol/g)	4.22 ± 0.29^*A*^	9.44 ± 0.43^*B*^
Hepatic TC (mmol/g)	1.64 ± 0.15^*A*^	3.30 ± 0.31^*B*^

*^1^Values are presented as the mean ± SD (*n* ≥ 10). Values with different capital letter superscripts mean highly significant difference between different groups (*P* ≤ 0.01). ^2^Liver index (‰) = humid weight of liver/body weight; TG, triglyceride; TC, total cholesterol.*

The PeakAnalyzer^3^ tool was used to scan the chicken genome and identify functional elements, proximal or distal to the transcript start site (Salmon-Divon et al., 2010). H3K27ac-enriched merged regions were considered distal putative enhancers if region boundaries were at a distance of over 1 kb from the transcription start site (TSS) following the similar discipline set by the previous report (Shen et al., 2012). Hypergeometric Optimization of Motif EnRichment (HOMER) (Heinz et al., 2010), a suite of tools for ChIP-Seq data analysis and motif discovery, was adopted to understand H3K27ac enrichment of distal and proximal regions across the genome and identify the transcription factor (TF) binding motifs associated with FLHS in chickens by running the command findMotifsGenome.pl.

To identify FLHS-associated differentially acetylated regions between healthy and FLHS chickens (Figure 4C), we followed a protocol set by previous reports (Nord et al., 2013) and quantified peak coverage based on read counts from the BAM files for peak read depth using the samtools (version 1.2) “bedcov” utility (Li et al., 2009). Empirical coverage was estimated by comparing coverage within 20-bp bins across one megabase of mappable chicken reference genome (Ensembl) sequence. ChIP and input sample coverage were normalized by total mapped read count and peak length, and input coverage was subtracted from ChIP coverage to limit the effects of fragmentation bias. We then used the DESeq2 R package (Love et al., 2014) to obtain differential H3K27ac regions between healthy and FLHS samples according to the normalized peak coverage. Regions with a *P*-value of less than 0.05 and |log2foldchange| ≥ 1 were considered as differential peaks.

### Identification of the Candidate Target Genes Regulated by Differential H3K27ac Peaks

To predict candidate targets regulated by differential H3K27ac peaks, we computed the correlation between the normalized counting reads of each differential peak and gene expression level (normalized as FPKM) within the same chromosomes (Takahashi et al., 2015). Correlated peak–gene pair was defined with Pearson correlation coefficient higher than 0.8 and *P*-value lower than 0.01. Then, we selected top 100 poorly and highly acetylated peak-associated genes by ordering both Cor and *P*-value first and then screen out the top 100 peak-associated genes for further functional enrichment analyses.

### Gene Ontology and Pathway Enrichment Analyses

Functional enrichment analyses were separately conducted for significantly hyper- and hypo-acetylated peaks (*P*-value ≤ 0.05). We then used DAVID^4^ (Bindea et al., 2009), PANTHER^5^ (Mi et al., 2013), and KEGG^6^ (a database resource for understanding high-level functions and utilities of the biological system) to identify overrepresented GO terms and pathways of the differentially expressed genes (DEGs), accompanied by H3K27ac changes in the same direction. GO terms with a corrected *P*-value of less than 0.05 were considered significantly enriched by DEGs.

### ChIP-qPCR and RT-PCR Validation

ChIP DNA was prepared using the SimpleChIP^®^ Plus Enzymatic Chromatin IP Kit (Magnetic Beads, 9005) with 500 μg chromatin from the same six samples used in RNA-seq and 5 μg anti-H3K27ac antibody (Active motif, 39133), following protocols from https://www.encodeproject.org/about/experiment-guidelines/ and https://www.animalgenome.org/community/FAANG. We adopted ChIP-qPCR to quantify ChIP DNA concentrations from samples in real time by analyzing fluorescent signal intensities that are proportional to the amount of amplicon after completing the ChIP assay and sample purification. Then, we firstly calculated the percent of input for each ChIP: %Input = 2^[–Δ^ ^*Ct*^ ^(*normalized* ChIP)]^, then normalized the positive locus ΔCt values to negative locus (ΔΔCt) by subtracting the ΔCt value obtained for the positive locus from the ΔCt value for negative locus: (ΔΔCt = ΔCt_*positive*_ - ΔCt_*negative*_), and finally, we calculated the fold enrichment of the positive locus sequence in ChIP DNA over the negative locus: fold enrichment = 2^Δ^ ^Δ^ ^*Ct*^.

RNA was reverse-transcribed into first-strand cDNA with Moloney Murine Leukemia Virus transcriptase (Promega, United States) and oligo (dT) (TaKaRa, Japan) using 2 μg of total RNA. The mRNA expression level of *PCK1*, *APOA1*, *FGF7*, and *KIT* genes were quantified by real-time PCR using a LightCycler 480 instrument with the LightCycler 480 SYBR Green I Master Mix (Roche, United States). For RT-PCR amplification, cDNA was pre-denatured at 95°C for 10 min, followed by 40 cycles of 95°C for 30 s and 60°C for 1 min. The relative expression level of the target gene was normalized to that of the housekeeping gene β-actin by the 2^–Δ^ ^Δ^ ^*CT*^ method.

## Results

### Pathological and Histopathological Differences Between the Liver Tissues From the FLHS and Normal Individuals

**FIGURE 1:**
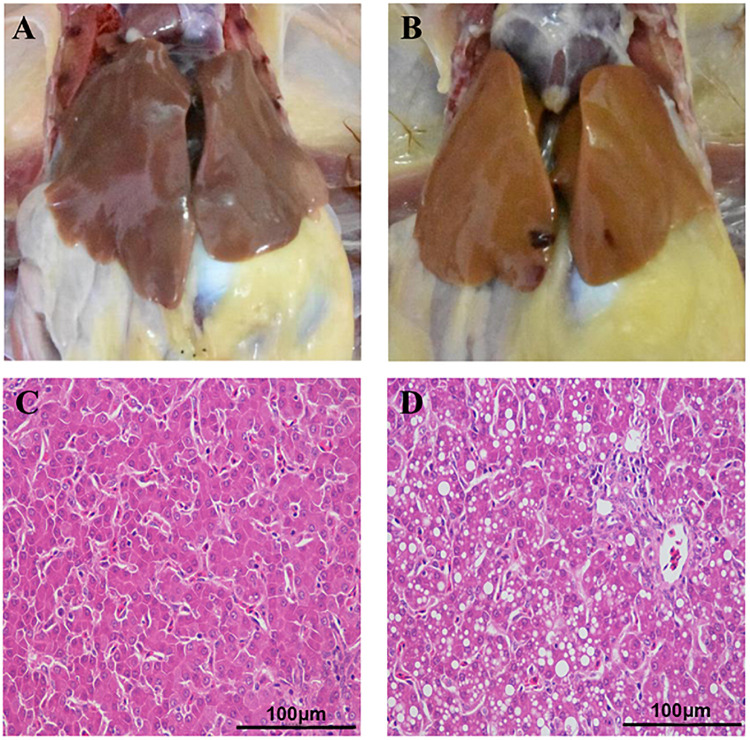
Anatomical and histopathological images of the liver in chickens. **(A)** Liver tissue of healthy chickens. **(B)** Liver tissue of FLHS chickens. **(C)** Histopathological section of the liver from the healthy group. **(D)** Histopathological section of the liver of the FLHS group. Scale bars = 100 μm.

Fatty liver hemorrhagic syndrome-affected chickens had large, friable, and soft livers in contrast to healthy hens (Figure 1A), and the color varied from yellow to orange (Figure 1B), which is consistent with previous reports (Spurlock and Savage, 1993; Rozenboim et al., 2016; Zhu et al., 2020). Similar to the results we previously published (Zhu et al., 2020), there was no statistical difference in body weight between hens of the two groups, but the liver index of the FLHS group was significantly (*P* ≤ 0.01) higher than that of the healthy group (Table 1). Further, the concentrations of hepatic triglycerides (TG) and total cholesterol (TC) were much higher (*P* ≤ 0.01) than in healthy individuals (Table 1). In addition, as H&E staining showed, the hepatocytes in the healthy group displayed regular hepatic sinusoids and normal hepatic cords (Figure 1C). However, the histology of the livers in the FLHS group demonstrated liver lesions, with micro-vesicular steatosis (a lot of hepatocytes containing vacuoles of variable sizes, i.e., fat droplets) (Figure 1D).

### Transcriptomic Differences Between the Healthy and FLHS Chickens

**FIGURE 2:**
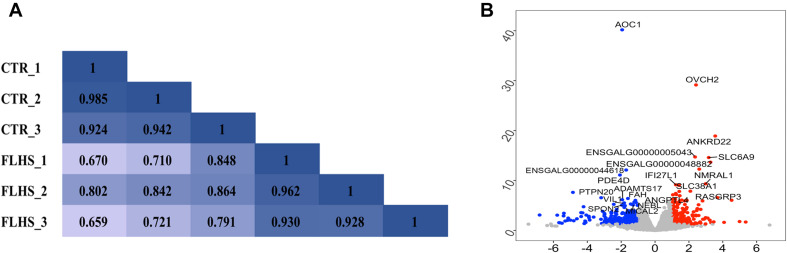
Distinct transcriptional signature between healthy and FLHS chickens. **(A)** Pearson correlation coefficient for each pair of biological replicates in RNA-Seq. **(B)** Volcano plot of top significant differentially expressed genes between healthy and FLHS chickens. The red and blue dot indicates upregulated gene and downregulated gene, respectively.

To reveal the global relationship between the liver epigenome and transcriptome in healthy and FLHS chickens, we firstly used RNA-Seq and obtained about 46.2 million (37.9∼53.8M) 150-bp paired-end reads for each sample. On average, 43.8 million (36.3∼50.5M) unique reads per sample were mapped to the chicken reference genome, and only ∼2 million were classified as improper pairs (Supplementary Table 2). A reasonable explanation of the amount of improperly mapped reads is the poor quality of the chicken genome assembly. We then investigated sample heterogeneity between healthy and FLHS samples by calculating Pearson correlation coefficients between the biological replicates. As shown in Figure 2A, the correlation coefficients between samples within each group (control or FLHS) were higher than those between groups, suggestive of the high biological reproducibility of our RNA-Seq data to be used for further analyses.

To identify differentially expressed genes between the healthy and FLHS chickens, we adopted the R package DESeq2 (Love et al., 2014) for read count normalization and DEG identification. In total, 443 DEGs were identified with a | log2 (fold change)| ≥ 1 and an adjusted *P*-value ≤ 0.05 (Supplementary Figure 1). Among these DEGs, 151 were upregulated and 292 were downregulated in FLHS chickens compared to healthy hens. The top 10 upregulated and downregulated genes are indicated in Figure 2B, including *OVCH2* (Wang and Ma, 2019), *ANKRD22* (Yin et al., 2017), *ENSGALG00000005043* (Moreira et al., 2018), *SLC6A9* (Alfadhel et al., 2016), *NMRAL1* (Garciandia and Suarez, 2013), *RASGRP3* (Oh-hora et al., 2003), *SLC38A1* (Kondoh et al., 2007), *ANGPTL4* (La Paglia et al., 2017; Singh et al., 2018), *AOC1* (Iffiu-Soltesz et al., 2010), *PDE4D* (Yun et al., 2016), *ADAMTS17* (Mead and Apte, 2018), *VIL1* (Xieraili et al., 2012; Roy et al., 2018), *FAH* (Grompe, 2017), and *SPON2* (Zhang Y. L. et al., 2018), which have been reported as involved in the regulation of inflammatory processes and lipid metabolism (Table 2).

**TABLE 2:** The top 10 up- and downregulated genes identified by RNA-Seq in FLHS chickens.

Genes	log2FoldChange	*P*-value	*P*-adj	Regulation	Functions
*OVCH2*	2.42	7.3E-30	5.4E-26	Up	Metabolism and transportation of amino acids
*ANKRD22*	3.56	1.2E-19	6.0E-16	Up	Inflammatory response and apoptosis
*ENSGALG00000005043*	2.36	1.9E-15	7.1E-12	Up	Cholesterol homeostasis and lipid metabolic process
*SLC6A9*	3.17	2.5E-15	7.4E-12	Up	Neurotransmitter and amino acid transport
*ENSGALG00000048882*	2.61	5.3E-13	1.1E-09	Up	/
*NMRAL1*	2.99	4.5E-10	6.7E-07	Up	Immune response and apoptosis
*IFI27L1*	1.23	7.3E-10	9.8E-07	Up	Apoptotic process
*RASGRP3*	2.08	1.4E-08	1.6E-05	Up	Ras protein signal transduction and immune response
*SLC38A1*	1.43	1.6E-08	1.6E-05	Up	Amino acid transporter and energy metabolism
*ANGPTL4*	1.25	6.6E-08	5.8E-05	Up	Glucose and lipid metabolism
*AOC1*	−1.94	6.6E-41	9.8E-37	Down	Lipid metabolism
*ENSGALG00000044618*	−1.71	7.9E-13	1.5E-09	Down	/
*PDE4D*	−2.07	8.0E-12	1.3E-08	Down	Phosphodiesterase activity and inflammatory response
*ADAMTS17*	−1.94	7.0E-07	4.0E-04	Down	Body fat distribution and metallopeptidase activity
*VIL1*	−1.83	3.0E-06	1.1E-03	Down	Inflammatory response
*FAH*	−1.44	5.1E-06	1.5E-03	Down	Immune response and type II diabetes mellitus
*PTPN20*	−2.44	5.9E-06	1.7E-03	Down	Proliferation, differentiation, migration, and survival
*MICAL2*	−1.38	6.1E-06	1.7E-03	Down	Cell differentiation and migration and angiogenesis
*NEBL*	−1.29	8.0E-06	2.1E-03	Down	Organelle organization and tropomyosin binding
*SPON2*	−1.96	8.9E-06	2.3E-03	Down	Immune responses

### Genome-Wide Profiling of H3K27ac Mark in the Healthy and FLHS Chickens

**FIGURE 3:**
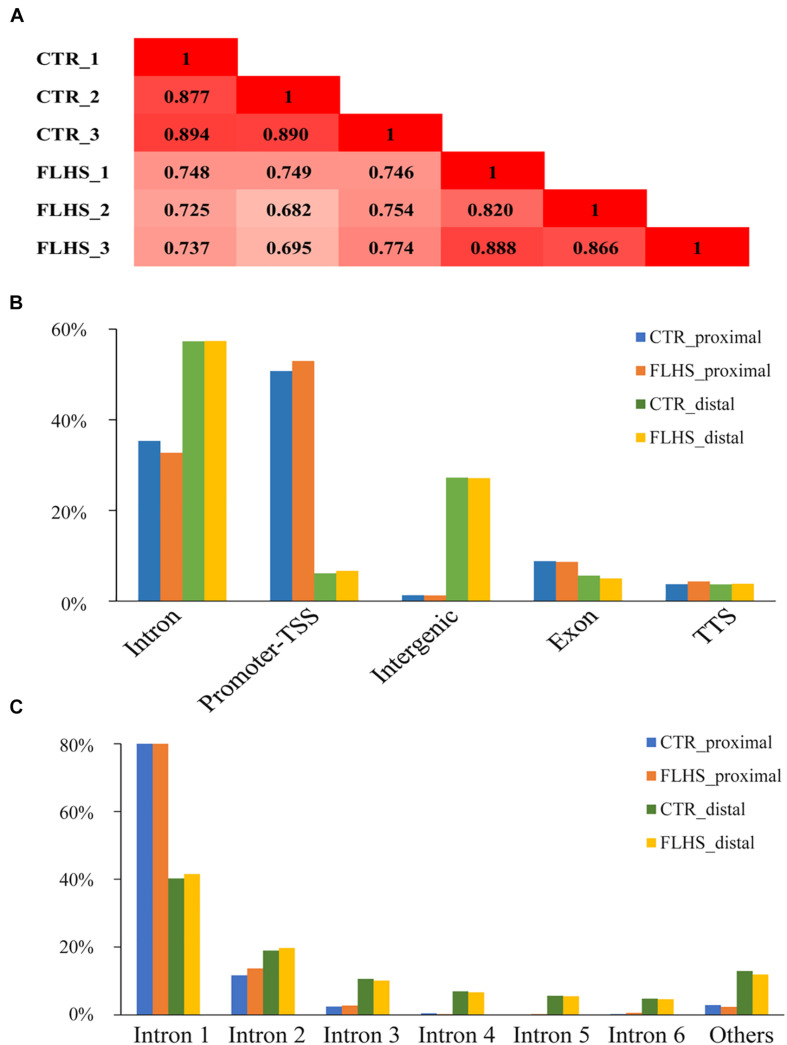
Mapping *in vivo* H3K27ac mark across genomic features via ChIP-Seq. **(A)** Pearson correlation coefficient for each pair of biological replicates in ChIP-Seq. **(B)** Breakdown on proximal and distal enrichment across genome in the healthy and FLHS. **(C)** The distribution of the H3K27ac peaks among each intron.

To examine the genome-wide *cis-*regulatory profiles of healthy and FLHS chickens, we adopted the H3K27ac epigenetic mark, a histone modification associated with active enhancers and promoters (Creyghton et al., 2010; Rada-Iglesias et al., 2011). We generated high-quality H3K27ac ChIP-seq data using postmortem liver tissues dissected from three healthy and three FLHS chickens (Supplementary Figure 2). For each ChIP or input sample, 35.5 million (29.4∼49.4M) 50-bp single-end reads were obtained. On average, 32.2 million (26.5∼44.9M) reads per sample were uniquely mapped to the chicken reference genome Gallus_gallus-6.0 (Ensembl) via BWA (Abuin et al., 2015; Supplementary Table 2). We then calculated Pearson’s correlation coefficients between samples to examine the reproducibility of our ChIP-seq assays. Pearson’s correlation analysis indicated that samples were clustered closely by treatment (control or FLHS), confirming high biological reproducibility data for further functional analysis (Figure 3A).

**FIGURE 4:**
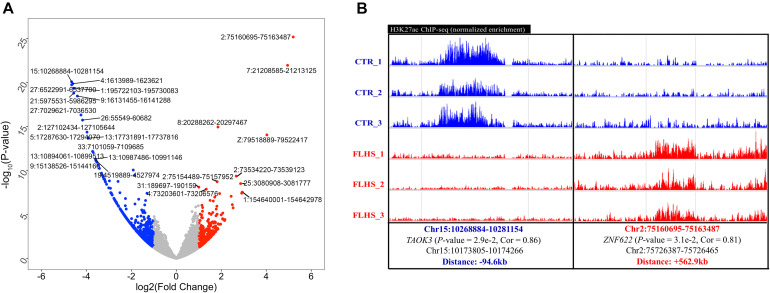
Differential H3K27ac peaks associated with FLHS in the liver. **(A)** Top 10 significant differential H3K27ac peaks between healthy and FLHS chickens. **(B)** Representative validation of differential peaks exhibiting distinct activity of the H3K27ac signal across healthy and FLHS chickens.

We identified peaks using MACS2 to determine H3K27ac enrichment loci across the genome (*q*-value = 1e-5) (Zhang et al., 2008). Following a protocol set by previous reports (Nord et al., 2013; Villar et al., 2015), we separated the H3K27ac-enriched regions into putative distal peaks, defined as regions positioned at least 1-kb away from known TSSs, and proximal regions that were within 1 kb of or overlapped TSSs. Supplementary Figure 2 shows a schematic overview of the analysis. In total, we identified 34,424 high-confidence H3K27ac peaks, including 2,045 regions that were proximal to known TSSs and 32,379 distal regions. We then used the HOMER software (Heinz et al., 2010) to understand the enrichment of putative distal and proximal peaks in each of the two groups. The larger proportion of putative distal peaks fell into intron (∼57.3% in controls and ∼57.4% in FLHS) and intergenic chromosomal regions (∼27.2% in controls and ∼27.1% in FLHS), while putative proximal peaks were mostly located in the promoter TSS region (∼50.8% in controls and ∼53.0% in FLHS) (Figure 3B), which was in agreement with the fact that putative proximal peaks were more likely to overlap with transcript start sites and promoters (Villar et al., 2015). In contrast, distal peaks had a tendency to fall into intron regions. In addition, both in controls and the FLHS group, a larger proportion of H3K27ac peaks overlapped with the first intron of corresponding genes (∼81.3% of the proximal and ∼40.9% of the distal) (Figure 3C), suggestive of the important regulatory role of sequences located in the first intron of genes. Taken together, these results revealed the landscape of H3K27ac modifications and the proximal and distal *cis-*regulatory element distribution of these regions.

### FLHS-Associated Differential H3K27ac Peaks in the Liver

We adopted the DESeq2 R package (Love et al., 2014) to obtain differential H3K27ac regions between healthy and FLHS samples. A total of 1,321 (4.1%) of the 32,379 peaks were characterized as FLHS-associated differential acetylation with a | log2 (fold change)| ≥ 1 and a *P*-value ≤ 0.05 (Supplementary Figure 3). Further, a significant enrichment of hypo-acetylated FLHS-associated peaks (894 or 2.8%) compared to hyper-acetylated FLHS-associated peaks (427 or 1.3%) was observed. The top 10 poorly and highly acetylated peaks are labeled in Figure 4A and Supplementary Table 3. To further validate the differential H3K27ac peaks between FLHS chickens and controls, we obtained the normalized signal of locations and putative associated target genes (Figure 4B and Supplementary Figure 4). For instance, an enhancer (Chr15:10268884-10281154) with higher H3K27ac enrichment in the control was related to *TAOK3* (*P*-value = 2.9e-2, Cor = 0.86), a gene associated with immune processes (Hammad et al., 2017). In contrast, a region (Chr2: 75160695-75163487) with increased hyper-acetylation in FLHS was identified near *ZNF622*, consistent with the known role of this gene in hepatic steatosis (Wu et al., 2018b). Together, these results reveal the widespread dysregulation of histone acetylation in the liver of FLHS chickens. In addition, we found that the distance between the differential peaks and associated genes were 94.6 and 562.9 kb (Supplementary Figure 4), respectively, which confirmed the enhancers are distal regulatory elements and may “skip” neighboring genes to regulate more “physically distant” ones (Andersson et al., 2014).

### Correlations in Activity Profiles Link Differential H3K27ac Peaks to Target Genes

**FIGURE 5:**
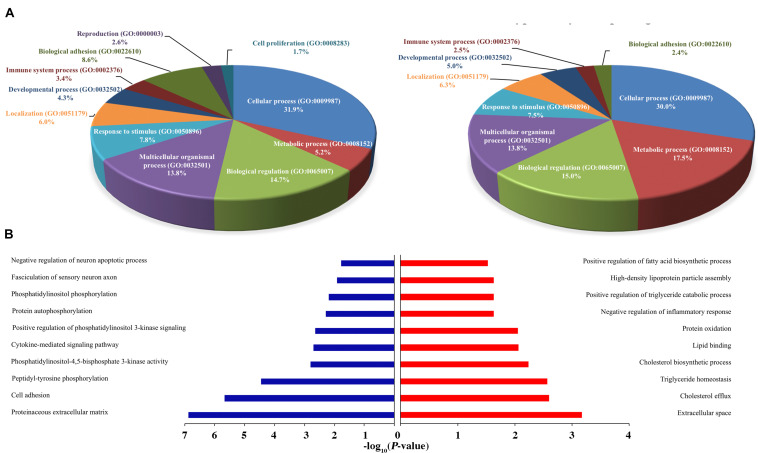
Functional annotation of H3K27ac ChIP-Seq and RNA-Seq between healthy and FLHS groups. **(A)** Functional enrichment of biological processes of putative target genes associated with differentially poorly and highly acetylated peaks. **(B)** Regulation terms of putative target genes of differentially poorly and highly acetylated peaks.

In order to assess the association between epigenetic changes and transcript levels, we integrated ChIP-Seq and RNA-Seq data and computed the correlation between activities of differential peaks and abundance of nearby transcripts within a 1-Mb region (Takahashi et al., 2015). We identified 4,204 significant peak–gene correlations with a Cor (peak–gene correlation) ≥0.8 and a *P-*value ≤ 0.01, including 438 and 3,764 correlations in the control and FLHS groups, respectively (Figure 5). Intriguingly, we also found that the number of negative peak–gene correlations was larger than that of positive peak–gene correlations for FLHS hyper-acetylated peaks (Supplementary Figure 5A). Compared to FLHS hypo-acetylated peak–genes (Supplementary Figure 5B), the frequency of peak–gene correlations within a region of 100 kb of the hyper-acetylated peaks was higher than beyond this region, showing that the frequency of both positive and negative correlations decayed with distance (Supplementary Figure 5C). In addition, the average distance between the peak and associated genes was 455.7 kb, which confirmed the active enhancer properties of H3K27ac, namely, that it can regulate genes without distance restrictions and may “skip” neighboring genes to regulate more “physically distant” ones (Creyghton et al., 2010; Pennacchio et al., 2013; Andersson et al., 2014; Marzi et al., 2018).

### TF Binding Motif Analysis of Differential H3K27ac Peaks

As TF binding motifs play an essential role in triggering epigenetic reprogramming, they may be abundant in regulatory regions that undergo epigenetic changes due to environmental stimuli (Kheradpour and Kellis, 2014; Nammo et al., 2018). Herein, we adopted HOMER^7^ to characterize the sequence motifs for DNA-binding proteins in differentially enriched H3K27ac regions in FLHS samples compared to controls. In total, 922 known TF binding motifs were enriched by differential H3K27ac peaks between healthy and FLHS chickens. Of those, 10 significantly enriched motifs (*P*-value ≤ 0.01) are listed in Table 3, including those for ERG, FOXA1, GABPA, FLI1, ETV1, PHA-4, ELF1, FOXO1, ETS1, and ETV2, factors reported to be involved in the regulation of inflammatory processes and metabolic homeostasis. Among them, ERG (Schafer et al., 2015), GABPA (Yuan et al., 2018), FLI1 (Chen et al., 2018), ETV1 (Baena et al., 2013; Heeg et al., 2016), ELF1 (Seifert et al., 2019), and ETS1 (Zhang Y. et al., 2018) are transcription factors associated with the immune system. Together, these results suggest that DNA-binding proteins involved in FLHS-induced epigenomic regulation are mainly transcription factors associated with the immune system and metabolic homeostasis. This observation is in accordance with the fact that transcription factors can serve as direct transcriptional activators and induce epigenetic changes in regulatory regions of the genome (Sugiaman-Trapman, 2018).

**TABLE 3:** The enriched TF binding motifs of FLHS differential H3K27ac peaks.

Rank	Name	Motif	*P*-value	*q*-value	# Target Sequences with Motif	% of Targets Sequences with Motif	Function
1	ERG	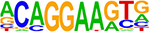	1.0E-04	1.3E-02	227	17.18%	Angiogenesis, inflammation, and apoptosis
2	FOXA1	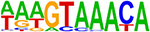	1.0E-04	1.3E-02	206	15.59%	Metabolism and glucose homeostasis
3	GABPA	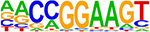	1.0E-04	2.1E-02	118	8.93%	Mitochondrial function, innate and acquired immunity
4	FLI1	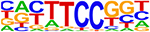	1.0E-03	3.2E-02	146	11.05%	Development of pre-T cell lymphoblastic leukemia/lymphoma
5	ETV1	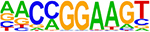	1.0E-03	3.2E-02	178	13.47%	Cell proliferation, apoptosis, and differentiation
6	PHA-4	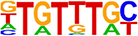	1.0E-03	3.7E-02	500	37.85%	Excessive lipid accumulation, energy metabolism
7	ELF1	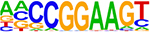	1.0E-03	3.7E-02	59	4.47%	Antiviral immune response
8	FOXO1	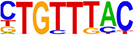	1.0E-03	4.5E-02	293	22.18%	Metabolism regulation, disease prevention
9	ETS1	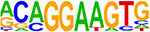	1.0E-03	4.5E-02	145	10.98%	Immune homeostasis
10	ETV2	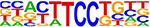	1.0E-03	4.7E-02	125	9.46%	Hematopoiesis and vasculogenesis

### Functional Enrichment of Differentially Expressed Genes That Were Accompanied by H3K27ac Changes

To conduct functional enrichment analysis of acetylic variation, we further identified the top 100 poorly and highly acetylated peak-associated genes, revealing overall increases and decreases in *cis-*regulatory activity, respectively (Please see section “Materials and Methods” for more details). We then used PANTHER (see text footnote 5) to understand the relevant biological processes of these differential peak-associated target genes. Remarkably, genes were overrepresented in multiple biological processes, such as “Cellular process (GO: 0009987),” “Metabolic process (GO: 0008152),” “Biological regulation (GO: 0065007),” “Response to stimulus (GO: 0050896),” and “Immune system process (GO: 0002376),” indicating that differentially acetylated peaks may trigger the expression of genes associated with these processes during the development of FHLS in chickens. Interestingly, FLHS hyper-acetylated peak-associated genes were more enriched in metabolic process, accounting for 17.5% compared to 5.2% for hypo-acetylated peak-associated genes (Figure 5A). For immune system processes, hypo-acetylated peak-associated genes (3.4%) were slightly more than FLHS hyper-acetylated peak-associated genes (2.5%). These results suggested that upregulated peak-associated genes may be more involved in metabolic processes, while downregulated peak-associated genes were more linked to other cellular processes, including immune processes. To further search for significantly overrepresented gene ontology terms associated with poorly and highly acetylated peak-associated genes, functional annotation was also performed using DAVID (see text footnote 4). Terms for the regulation of cholesterol and lipid metabolic processes were significantly enriched (*P-*value ≤ 0.01, Figure 5B) in the FLHS group, in consistence with results of the PANTHER-based analysis (Figure 5A).

### Overrepresented Pathways of Differential Peak–Genes

We tried to better understand pathways that were overrepresented by all differential peak-associated genes between control and FLHS chickens by using DAVID (see text footnote 4) and the KEGG database (see text footnote 6). In total, five significant pathways [peroxisome proliferator-activated receptor (PPAR) signaling pathway, fat digestion and absorption, PI3K-Akt signaling pathway, Rap1 signaling pathway, and the MAPK signaling pathway] were enriched (*P-*value ≤ 0.05) (Table 4), including peak-associated genes *PCK1* (Chr20:11003689-11003961) (Millward et al., 2010), *APOA1* (Chr24:5089487-5090379) (Li et al., 2018), *ANGPTL4* (Chr28:1779594-1787257) (Oteng et al., 2019), *FABP1* (Chr4:86731254-86735175) (Rodriguez Sawicki et al., 2017), *NTRK2* (ChrZ:41171358-41171800) (Geibel et al., 2014), *FGF7* (Chr10:11491528-11493116) (Itoh et al., 2016), *PDGFRA* (Chr4:65059385-65060498) (Awuah et al., 2013), and *KIT* (Chr4:65997303-65998627) (Rice et al., 2018), which are related to lipid metabolism, glucose homeostasis, and immune processes (Table 5). Of note, the most significantly enriched pathway, the PPAR signaling pathway, has three branches: PPARα, PPARβ/δ, and PPARγ, which are involved in the regulation of peroxisome proliferation, hepato-carcinogenesis, fatty acid metabolism, lipid homeostasis, adipocyte differentiation, and glucose metabolism (Supplementary Figure 6). Among those, PPARα is a nutrient sensor, which allows for the adaptation to fatty acid catabolism, lipogenesis, and ketone body synthesis in response to feeding and starvation in rodent models during systemic inflammation, atherosclerosis, and non-alcoholic steatohepatitis (NASH) (Pawlak et al., 2015). PPARβ/δ is a ligand-activated transcription factor, involved in the regulation of lipid metabolism, glucose homeostasis, and insulin sensitivity (Zarei et al., 2017). PPARγ signaling has also been implicated in the control of lipid homeostasis, macrophage function, and immunity through insulin-sensitive activation, lipid storage, metabolic regulation, and inflammatory mediators termed adipokines (Tontonoz and Spiegelman, 2008). Taken together, targeting the PPAR signaling pathway may improve insulin sensitivity, prevent lipid accumulation, and reduce liver damage during FLHS inflammatory attacks in chickens.

**TABLE 4:** Top canonical pathways that are enriched by differentially peak–genes between FLHS-affected and non-affected chickens.

ID	Pathways	*P*-value	Associated peak–genes	Regulated	Function
hsa03320	PPAR signaling pathway	3.58E-03	*APOA1*, *FABP1*, *PCK1*, and *ANGPTL4*	Up	It involved in several physiological processes including modulation of cellular differentiation; development; metabolism of carbohydrates, lipids, and proteins; and tumorigenesis
hsa04975	Fat digestion and absorption	1.40E-02	*APOA4*, *APOA1*, and *FABP1*	Up	It is essential in the prevention of a calorie balance disorder and improving the pathological conditions of metabolic syndrome through improvement of obesity
hsa04151	PI3K-Akt signaling pathway	1.59E-02	*LAMA4*, *FGF7*, *PDGFRA*, *PDGFD*, and *KIT*	Down	It is critical in restricting pro-inflammatory and promoting anti-inflammatory responses in TLR-stimulated macrophages and has been considered as a negative regulator of TLR and NF-κB signaling in macrophages
hsa04015	Rap1 signaling pathway	2.18E-02	*FGF7*, *PDGFRA*, *PDGFD*, and *KIT*	Down	It plays a dominant role in the control of cell–cell and cell–matrix interactions by regulating the function of integrins and other adhesion molecules in various cell types. Rap1 also regulates MAP kinase (MAPK) activity in a manner highly dependent on the context of cell types
hsa04010	MAPK signaling pathway	3.53E-02	*FGF7*, *NTF3*, *NTRK2*, and *PDGFRA*	Down	It is essential in regulating many cellular processes including inflammation, cell stress response, cell differentiation, cell division, cell proliferation, metabolism, motility, and apoptosis

**TABLE 5:** Interested genes enriched in pathways which related with lipid metabolic, glucose homeostasis, and immune processes, etc.

Gene	Symbol	FoldChange	*P*-value	H3K27ac peak	Correlation	Regulated	Function
*PCK1*	Phosphoenolpyruvate carboxykinase 1	7.90	4.9E-02	Chr20:11003689-11003961	0.890	Up	This gene is a main control point for the regulation of gluconeogenesis and the expression can be regulated by insulin, glucocorticoids, glucagon, cAMP, and diet
*APOA1*	Apolipoprotein A1	3.09	2.9E-02	Chr24:5089487-5090379	0.887	Up	The encoded preproprotein promotes cholesterol efflux from tissues to the liver for excretion and is a cofactor for lecithin cholesterol acyltransferase (LCAT), an enzyme responsible for the formation of most plasma cholesteryl esters
*ANGPTL4*	Angiopoietin-like 4	2.38	6.6E-08	Chr28:1779594-1787257	0.983	Up	This gene encodes a glycosylated, which functions as a serum hormone that regulates glucose homeostasis, lipid metabolism, and insulin sensitivity
*FABP1*	Fatty acid binding protein 1	2.04	1.7E-03	Chr4:86731254-86735175	0.929	Up	This gene encodes the fatty acid binding protein found in the liver and plays an essential role in fatty acid uptake, transport, and metabolism
*NTRK2*	Neurotrophic receptor tyrosine kinase 2	7.21	1.1E-02	ChrZ:41171358-41171800	0.902	Down	This gene encodes a member of the neurotrophic tyrosine receptor kinase (NTRK) family and associates with obesity and mood disorders
*FGF7*	Fibroblast growth factor 7	5.55	2.0E-02	Chr10:11491528-11493116	0.974	Down	The protein encoded by this gene is a member of the fibroblast growth factor (FGF) family and involves in a variety of biological processes, including embryonic development, cell growth, morphogenesis, tissue repair, tumor growth, and invasion
*PDGFRA*	Platelet-derived growth factor receptor alpha	2.85	9.4E-04	Chr4:65059385-65060498	0.864	Down	This gene plays a role in organ development, wound healing, and tumor progression
*KIT*	Receptor tyrosine kinase	2.48	3.7E-02	Chr4:65997303-65998627	0.892	Down	It encodes a tyrosine kinase receptor and is part of signaling pathways that control multiple cellular processes, including cell proliferation, survival, and migration

### ChIP-qPCR and RT-PCR Validation of Peak-Associated Target Genes

**FIGURE 6:**
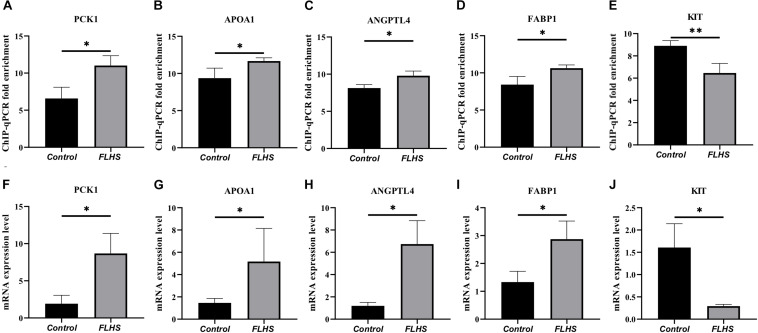
ChIP-qPCR and RT-PCR validation of peak-associated target genes. **(A–E)** ChIP-qPCR fold enrichment of *PCK1*, *APOA1*, *ANGPTL4*, *FABP1*, and *KIT* genes in FLHS pathological chickens relative to control individuals. **(F–J)** The mRNA expression levels of *PCK1*, *APOA1*, *ANGPTL4*, *FABP1*, and *KIT* genes in FLHS pathological chickens relative to control individuals. Expression levels were determined as the fold change in 2^–Δ^ ^Δ^ ^*Ct*^ levels relative to the control group with median expression set to 1. Significance was assessed by *t*-test, *P ≤ 0.05, **P ≤ 0.01.

To test the interested genes enriched in pathways that related with lipid metabolic, glucose homeostasis, and immune processes, we further performed ChIP-qPCR and RT-PCR to test the enrichment and mRNA expression levels of *PCK1*, *APOA1*, *ANGPTL4*, *FABP1*, and *KIT* genes in FLHS pathological chickens relative to control individuals. The ChIP-qPCR fold enrichment of *PCK1*, *APOA1*, *ANGPTL4*, and *FABP1* was significantly increased (*P* ≤ 0.05) in the FLHS samples (Figures 6A–D), in accordance with the mRNA expression level (Figures 6F–I), while *KIT* peak was significantly decreased (*P* ≤ 0.05) (Figure 6E), which is also consistent with our RNA-seq result (Figure 6J).

## Discussion

In this study, we describe the alterations of acetylic variation and the transcriptome landscape resulting from a standard and a high-energy, low-protein (HELP) diet. Intriguingly, as metabolic disorders such as obesity and liver steatosis often stem from the dysregulation of lipid homeostasis and immune pathways (Steinberg, 2007; Gurzov et al., 2016), our study also revealed that differentially acetylated peaks were involved in the regulation of a number of genes enriched in lipid metabolism, glucose homeostasis, and immune biological processes, including *PCK1*, *APOA1*, *ANGPTL4*, *FABP1*, *NTRK2*, *FGF7*, *PDGFRA*, and *KIT*. To the best of our knowledge, this is the first study to investigate the variation of H3K27ac marks in FLHS in chickens. Further, apart from identifying candidate genes and molecular pathways, we also provide a framework for future genome-wide studies of this modification.

In addition, our analyses revealed that diet-induced FLHS resulted in genome-wide epigenetic alterations and enrichment of TF binding motifs in H3K27ac regions, including the binding motifs for ERG, FOXA1, GABPA, FLI1, ETV1, PHA-4, ELF1, FOXO1, ETS1, and ETV2, which are factors reported to be involved in immune system processes and metabolic homeostasis in FLHS chickens, in accordance with the results of the functional enrichment analysis of candidate differentially expressed genes. For example, ERG, a transcription factor of the ETS family, plays key roles in the regulation of cell proliferation, differentiation, inflammation, and apoptosis (Schafer et al., 2015). PHA-4 (Panowski et al., 2007; Wu et al., 2018a), FOXA1 (Swinstead et al., 2016; Xing et al., 2018; Zhou et al., 2020), and FOXO1 (Xing et al., 2018) are transcription factors related to metabolic homeostasis. Wu et al. have revealed that PHA-4/FoxA functions as a sensor of nucleolar stress. The complex binds and transactivates the expression of the lipogenic genes, promoting subsequent lipid accumulation (Panowski et al., 2007; Wu et al., 2018a). Xing et al. (2018) discovered that Forkhead box transcription factor O1 (FOXO1) plays a role in response to cellular stimulation and maintenance of tissue homeostasis. This observation further confirmed the fact that TF motifs act as transcriptional activators and play a vital role in regulating transcriptional programs that control cellular behavior and disease phenotypes (Corradin and Scacheri, 2014; Sugiaman-Trapman, 2018).

**FIGURE 7:**
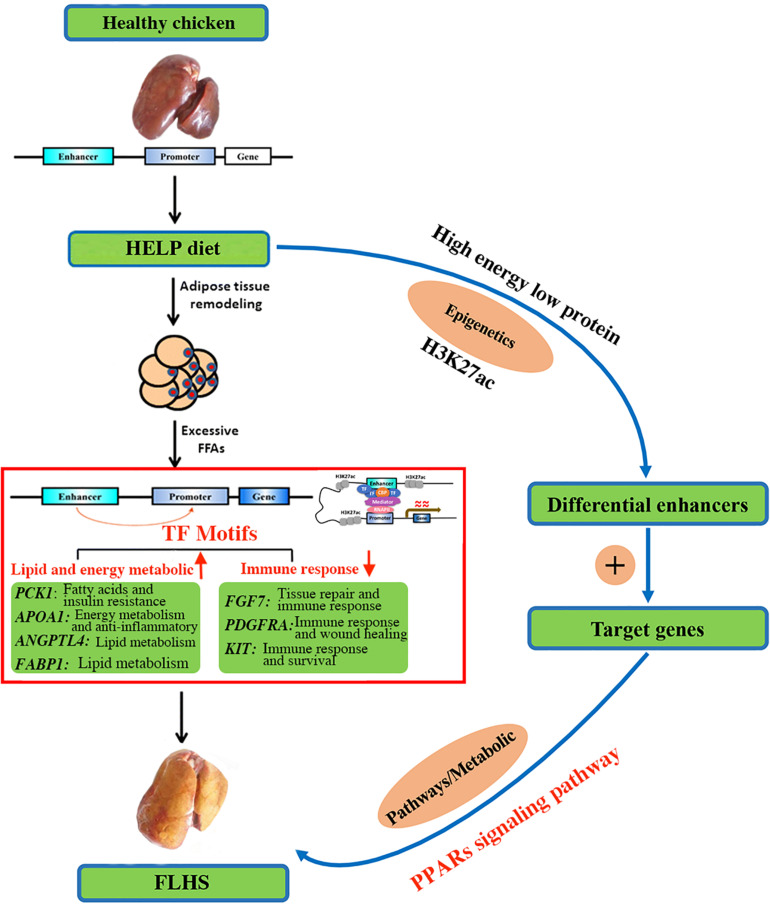
A proposed model on the epigenetic regulation mechanisms of histone H3K27 acetylation causing the FLHS in laying hens. Mediated by histone-modifying enzyme, HELP could have an impact on histone modification of H3K27ac and chromatin structure, leading to the dysregulation of genes related to lipid and energy metabolism (*PCK1*, *APOA1*, *ANGPTL4*, and *FABP1*) and immune response (*FGF7*, *PDGFRA*, and *KIT*), and PPAR signaling pathway, which may cause the excessive accumulation of fat in liver tissue and induce the development of the FLHS.

Given its close relationship with transcriptional activation through transcription factor binding, we highlighted target genes of FLHS-associated variation in H3K27ac. Most significantly enriched were the PPAR signaling pathways, which act as regulators of lipid and lipoprotein metabolism and glucose homeostasis. Based on the biological functions of candidate genes (Table 5) and previous studies of PPAR signaling, we proposed a model of the involvement of H3K27ac in the epigenetic regulation mechanism of FLHS in laying hens (Figure 7). In this model, the HELP diet may have an impact on histone modification of H3K27ac and chromatin structure, leading to the dysregulation of candidate genes related to lipid and energy metabolism (*PCK1*, *APOA1*, *ANGPTL4*, and *FABP1*), the immune system (*FGF7*, *PDGFRA*, and *KIT*), and PPAR signaling, which causes the excessive accumulation of fat in liver tissue and induces the formation of FLHS, supportive of the notion that changes in epigenetic modifications and transcriptional regulation are essential for phenotypic adaptation to environmental stimuli (Ling and Groop, 2009; Corradin and Scacheri, 2014; Carrer et al., 2017; Siersbaek et al., 2017; Marzi et al., 2018; Jahan et al., 2020). Thus, better understanding of the PPAR pathways and the functional roles of candidate genes in the context of FLHS are of great value and may enable us to develop new and specific therapies for FLHS-susceptible commercial laying hens.

It is important to note that the current study had a number of limitations. First, the number of tested samples was relatively small. In light of this, we have observed substantial differences between healthy and FLHS cases, including FLHS-associated regulatory alterations in genes and pathways that are known to play a role in the development of FLHS. Differential acetylated peaks of controls and FLHS chickens showed clear clustering, which implies that samples were mainly distinguished by a common molecular pathology of lipid homeostasis, energy metabolism, and immune function. In addition, the results of our TF binding motif analyses and functional enrichment analyses of genes associated with differentially acetylated peaks were in consistence with previous observations from larger numbers of samples. Second, H3K27ac, while a typical modification that regulates genomic function, can only provide relatively limited information about transcriptional activity (Kimura, 2013; Nammo et al., 2018). We found the differences in a number of genes annotated to differentially acetylated peaks and revealed their associated signaling pathways, supporting the notion that dysregulation of H3K27 acetylation and the PPAR signaling pathway are involved in the excessive accumulation of fat in liver tissue and the onset of lipid metabolic disorders (Chinetti et al., 2000; Beacon et al., 2020). Nevertheless, further experiments are still required to confirm the model and candidate genes.

This study provided evidence for the widespread H3K27ac dysregulation in the liver of FLHS-affected chickens and the enrichment of TF binding sequence motifs in response to disease. Notably, we identified that FLHS-associated variations in H3K27ac marks were associated with multiple known FLHS risk genes involved in lipid and energy metabolism (*PCK1*, *APOA1*, *ANGPTL4*, and *FABP1*) and immune function (*FGF7*, *PDGFRA*, and *KIT*). Further, we observed that the PPAR signaling pathway was the most significantly enriched in FLHS. In addition, we present a framework for integrating genome-wide studies of histone modifications and transcriptional regulation, both critical for the phenotypic adaptation to environmental stimuli, in the study of lipid metabolism disorders.

## Conclusion

This study provided evidence for widespread acetylic variation in the liver of FLHS-affected chickens and enrichment of sequence motifs showing changes in response to disease. Notably, we identified that FLHS-regulatory variable H3K27ac was associated with multiple known FLHS risk genes, which robustly associated with lipid and energy metabolism (*PCK1*, *APOA1*, *ANGPTL4*, and *FABP1*) and immune system (*FGF7*, *PDGFRA*, and *KIT*), and discovered the most significantly enriched signaling pathway of PPARs. We also presented a framework for genome-wide studies of histone modifications in lipid metabolic disorders, by integrating our data with results obtained from transcriptional regulation, which are both essential for phenotypic adaptation to environments.

## Data Availability Statement

The RNA-Seq and ChIP-Seq data of healthy and FLHS individuals analyzed for this study can be found at GSA (https://bigd.big.ac.cn/gsa/browse/CRA002527).

## Ethics Statement

The animal study was reviewed and approved by the Jiangxi Agricultural University and Anhui Medical University. Written informed consent was obtained from the owners for the participation of their animals in this study.

## Author Contributions

JH and JR conceived and designed the experiments. QZ, ZZ, CY, and YW performed the experiments. YZ, QZ, TJ, and FL analyzed the data. YZ, JH, and JR wrote and revised the manuscript. All authors read and approved the final manuscript.

## Conflict of Interest

The authors declare that the research was conducted in the absence of any commercial or financial relationships that could be construed as a potential conflict of interest.

## Footnotes

1 https://www.faang.org

2 https://www.r-project.org/

3 https://www.bioinformatics.org/peakanalyzer/

4 https://david-d.ncifcrf.gov/

5 http://www.pantherdb.org/

6 http://www.genome.jp/kegg/

7 http://homer.ucsd.edu/homer/motif/motifDatabase.html
