# Supplementary material for: Dysregulated H3K27 Acetylation Is Implicated in Fatty Liver Hemorrhagic Syndrome in Chickens
Source: Front Genet. 2021 Jan 11;11:574167. doi: 10.3389/fgene.2020.574167 (PMC7831272; doi:10.3389/fgene.2020.574167)
Supplement: Supplementary file 1 [file Data_Sheet_1.docx]

**SUPPLEMENTARY MATERIAL**

**Dysregulated H3K27 acetylation is implicated in fatty liver hemorrhagic syndrome in chickens**

The PDF file includes:
Four supplementary tables: Table S1 – S3
Onesupplementary figures: Figure S1 – S6

**Table S1. Ingredient and nutrient levels of the HELP diets for control and experimental samples**

**Table S2. Number of uniquely mapped reads for each ChIP-Seq and RNA-Seq samples**

**Table S3. The top 10 hypo- and hyper-acetylated FLHS-associated peaks in the liver.**

**Figure S1. Transcription profiles of 443 differentially expressed genes between healthy (N=292) and FLHS (N=151) (Threshold: fold change ≥ 2 & P-value ≤ 0.05). CTR, control; FLHS, Fatty liver hemorrhagic syndrome.**

**Figure S2. ChIP analysis workflow showing experimental (light blue) and computational steps (pink) and summary numbers.**

**Figure S3. Unsupervised hierarchical clustering of the differential H3K27ac peaks between healthy (N=894) and FLHS (N=427) chickens (Significance of differential peak activity was determined with fold change ≥ 2 & P-value ≤ 0.05).**

**Figure S4. Representative validation of differential peak-genes exhibiting distinct mRNA expression level across healthy and FLHS chickens.**

**Figure S5. Integrated analysis of H3K27ac CHIP-Seq and RNA-Seq between healthy and FLHS groups.** (A) The number of positive and negative peak-gene interaction in CTR and FLHS. (B) Distribution of peak–gene correlations against peak–gene distance in hypo-acetylated peaks. (C) Distribution of peak–gene correlations against peak–gene distance in FLHS hyper-acetylated peaks. And the frequency in the middle within 100kb were more condense than outside with far distance.

**Figure S6. Pathway of cytokine-cytokine receptor interaction.**

**Table S1. Ingredient and nutrient levels of the HELP diets for control and experimental samples**

| HELP diet | | Control group (%) | Experimental group (%) |
| --- | --- | --- | --- |
| Ingredient | Corn | 64 | 70 |
|  | Wheat bran | 2 | 1.2 |
|  | Soybean meal | 24 | 14.58 |
|  | Fat-soybean oil | - | 4.22 |
|  | Limestone | 8 | 8 |
|  | Premix* | 2 | 2 |
| Nutrient levels | Energy (Mcal/kg) | 11.21 | 12.97 |
|  | Crude protein (%) | 15.86 | 12 |
|  | P (%) | 0.51 | 0.46 |
|  | Arg (%) | 1.03 | 0.74 |
|  | Met (%) | 0.37 | 0.32 |
|  | Val (%) | 0.77 | 0.58 |
|  | Met + Cys (%) | 0.67 | 0.56 |

*Premix was composed of the following per kg diet: Cu, 2.50 mg; Fe, 20.00 mg; Zn, 17.50 mg; Mn, 15.00 mg; KI, 4.00 mg; Na2SeO3, 6.00 mg; CoCl, 2.50 mg; Met, 50.00 mg; pyridine chromium, 2.00 mg; vitamins, 15.00 mg; phytase, 10.00 mg; kininase, 7.50 mg; antioxidant, 2.00 mg; lycine, 15.00 mg; choline, 50.00 mg; salt, 200.00 mg; Ca3PO4, 500.00 mg and zeolite, 76.00 mg.

**Table S2. Number of uniquely mapped reads for each ChIP-Seq and RNA-Seqsamples**

| Method | Summary | CTR 1 | CTR 2 | CTR 3 | FLHS 1 | FLHS 2 | FLHS 3 |
| --- | --- | --- | --- | --- | --- | --- | --- |
| ChIP-Seq | Input total mapped reads | 38143239 | 32786514 | 39463710 | 37278526 | 31934221 | 31835207 |
|  | Input unique mapped reads | 34340031 | 29536314 | 35824731 | 33924170 | 28865558 | 29070995 |
|  | Input unique mapped ratio | 90.0% | 90.1% | 90.8% | 91.0% | 90.4% | 91.3% |
|  | Ac total reads | 38918254 | 29391267 | 49350432 | 34658196 | 33386686 | 28874167 |
|  | Ac unique mapped reads | 34952848 | 26465181 | 44857291 | 31836317 | 30226702 | 26342150 |
|  | Ac unique mapped ratio | 89.8% | 90.0% | 90.9% | 91.9% | 90.5% | 91.2% |
|  | H3K27ac peaks | 25742 | 21624 | 21227 | 19586 | 12450 | 16065 |
| RNA-Seq | Total mapped reads | 37916214 | 41630718 | 53766016 | 47037241 | 47336496 | 49475006 |
|  | Unique mapped reads | 36322622 | 39728789 | 50514127 | 44408233 | 45135599 | 47066323 |
|  | Unique mapped ratio | 95.8% | 95.4% | 94.0% | 94.4% | 95.4% | 95.1% |
|  | Multiple_mapped | 1.1% | 1.2% | 1.4% | 1.2% | 1.1% | 1.1% |

**Table S3. The top 10 hypo- and hyper-acetylated FLHS-associated peaks in the liver.**

| Rank | Chr. | Position (Start-End) | log_2_(FoldChange) | *P*-value | FDR |
| --- | --- | --- | --- | --- | --- |
| Hypoacetylated peaks | |  |  |  |  |
| 1 | 15 | 10268884-10281154 | -4.6575388 | 8.12E-21 | 6.54E-17 |
| 2 | 27 | 6522991-6537700 | -4.65339305 | 1.81E-20 | 8.75E-17 |
| 3 | 4 | 1613989-1623621 | -4.605046148 | 1.35E-20 | 8.17E-17 |
| 4 | 1 | 195722103-195730083 | -4.571723354 | 4.41E-20 | 1.78E-16 |
| 5 | 21 | 5975531-5986295 | -4.562802794 | 1.60E-19 | 5.52E-16 |
| 6 | 9 | 16131455-16141288 | -4.400548833 | 3.43E-19 | 1.04E-15 |
| 7 | 27 | 7029621-7036530 | -4.241317799 | 4.86E-17 | 1.31E-13 |
| 8 | 26 | 55549-60682 | -4.171321166 | 1.86E-16 | 4.50E-13 |
| 9 | 2 | 127102434-127105644 | -3.972064815 | 4.35E-15 | 8.76E-12 |
| 10 | 5 | 17287630-17294070 | -3.9199654 | 1.88E-14 | 3.25E-11 |
| Hyperacetylated peaks | |  |  |  |  |
| 1 | 2 | 75160695-75163487 | 5.198035017 | 6.51E-26 | 1.58E-21 |
| 2 | 7 | 21208585-21213125 | 4.94862823 | 1.10E-22 | 1.33E-18 |
| 3 | 8 | 20288262-20297467 | 1.854172623 | 1.15E-15 | 2.52E-12 |
| 4 | Z | 79518889-79522417 | 4.028058595 | 9.02E-15 | 1.68E-11 |
| 5 | 2 | 73534220-73539123 | 2.664304647 | 4.38E-10 | 3.65E-07 |
| 6 | 2 | 75154489-75157952 | 2.859906629 | 3.14E-09 | 1.90E-06 |
| 7 | 25 | 3080908-3081777 | 2.932978537 | 2.89E-08 | 1.52E-05 |
| 8 | 31 | 189697-190159 | 2.907031586 | 3.86E-08 | 1.86E-05 |
| 9 | 4 | 73203601-73206576 | 1.926639918 | 4.25E-08 | 1.98E-05 |
| 10 | 1 | 154640001-154642978 | 2.438608525 | 8.72E-08 | 3.83E-05 |

**Figure S1. Transcription profiles of 443 differentially expressed genes between healthy (N=292) and FLHS (N=151) (Threshold: fold change ≥ 2 & P-value ≤ 0.05). CTR, control; FLHS, Fatty liver hemorrhagic syndrome.**

**
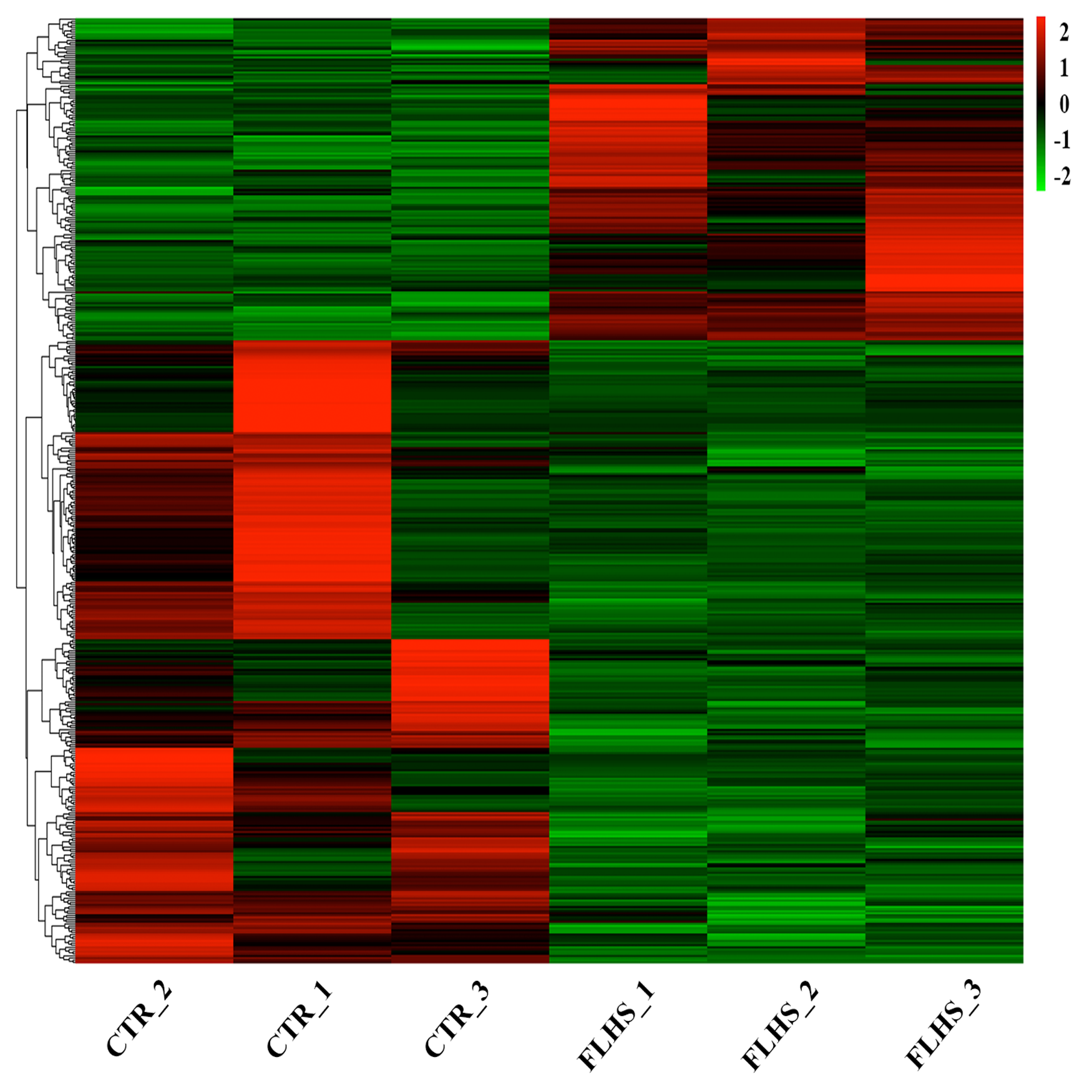
**

**Figure S2. ChIP analysis workflow showing experimental (light blue) and computational steps (pink) and summary numbers.**

**
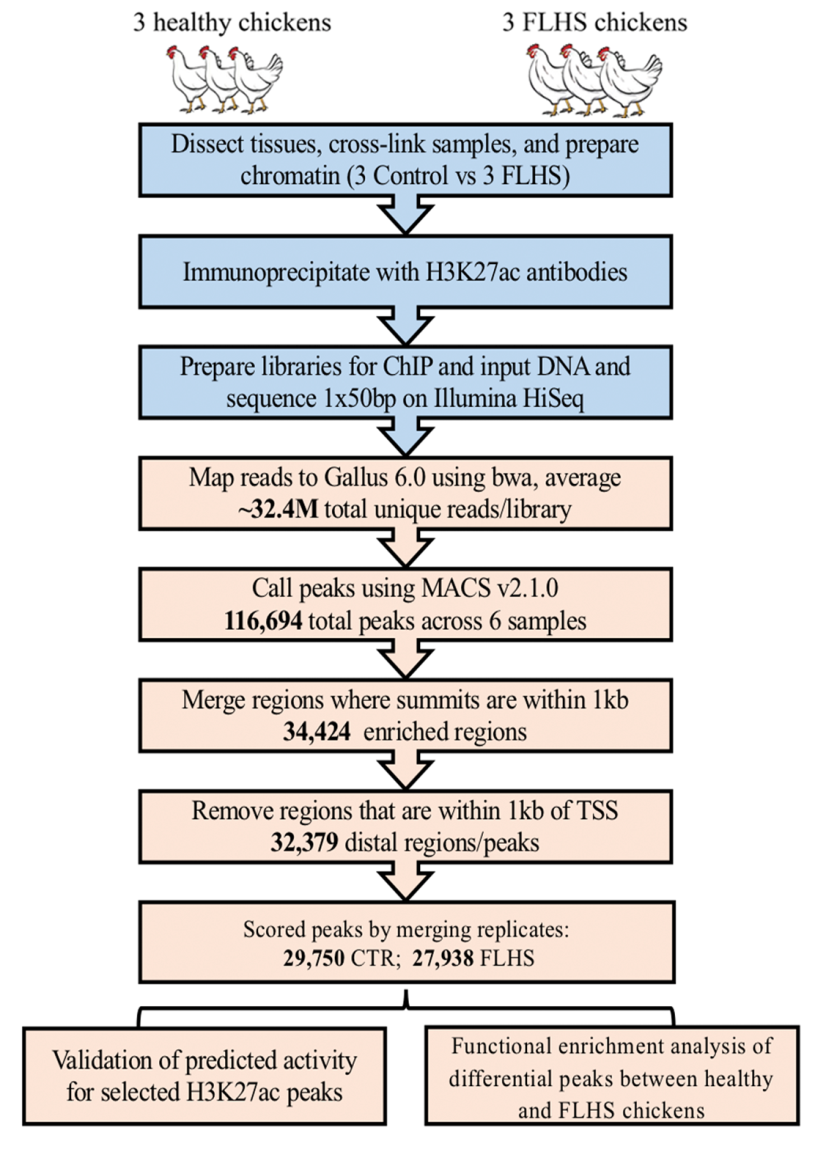
**

**Figure S3. Unsupervised hierarchical clustering of the differential H3K27ac peaks between healthy (N=894) and FLHS (N=427) chickens (Significance of differential peak activity was determined with fold change ≥ 2 & P-value ≤ 0.05).**

**
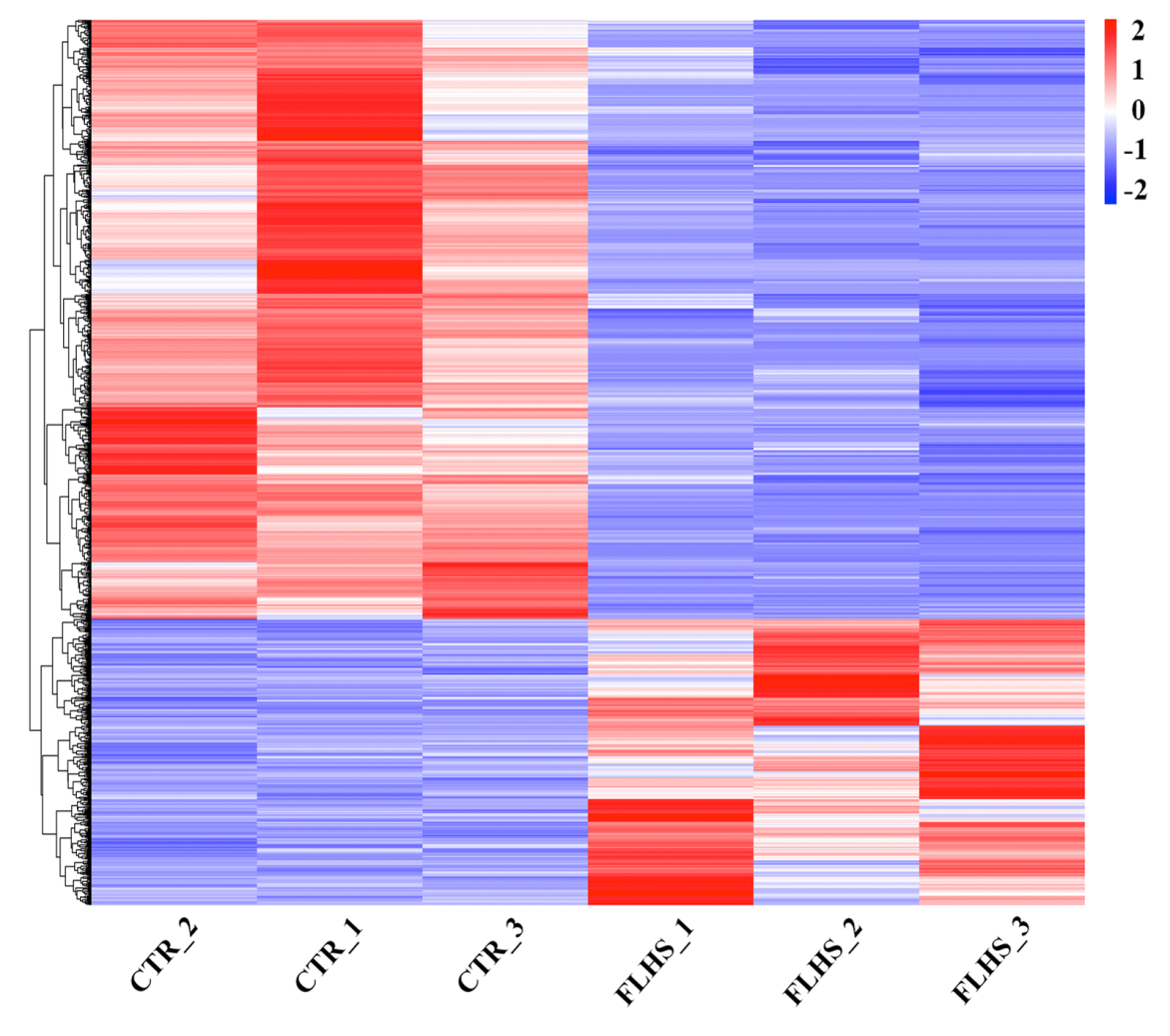
**

**Figure S4. Representative validation of differential peak-genes exhibiting distinct mRNA expression level across healthy and FLHS chickens.**

**
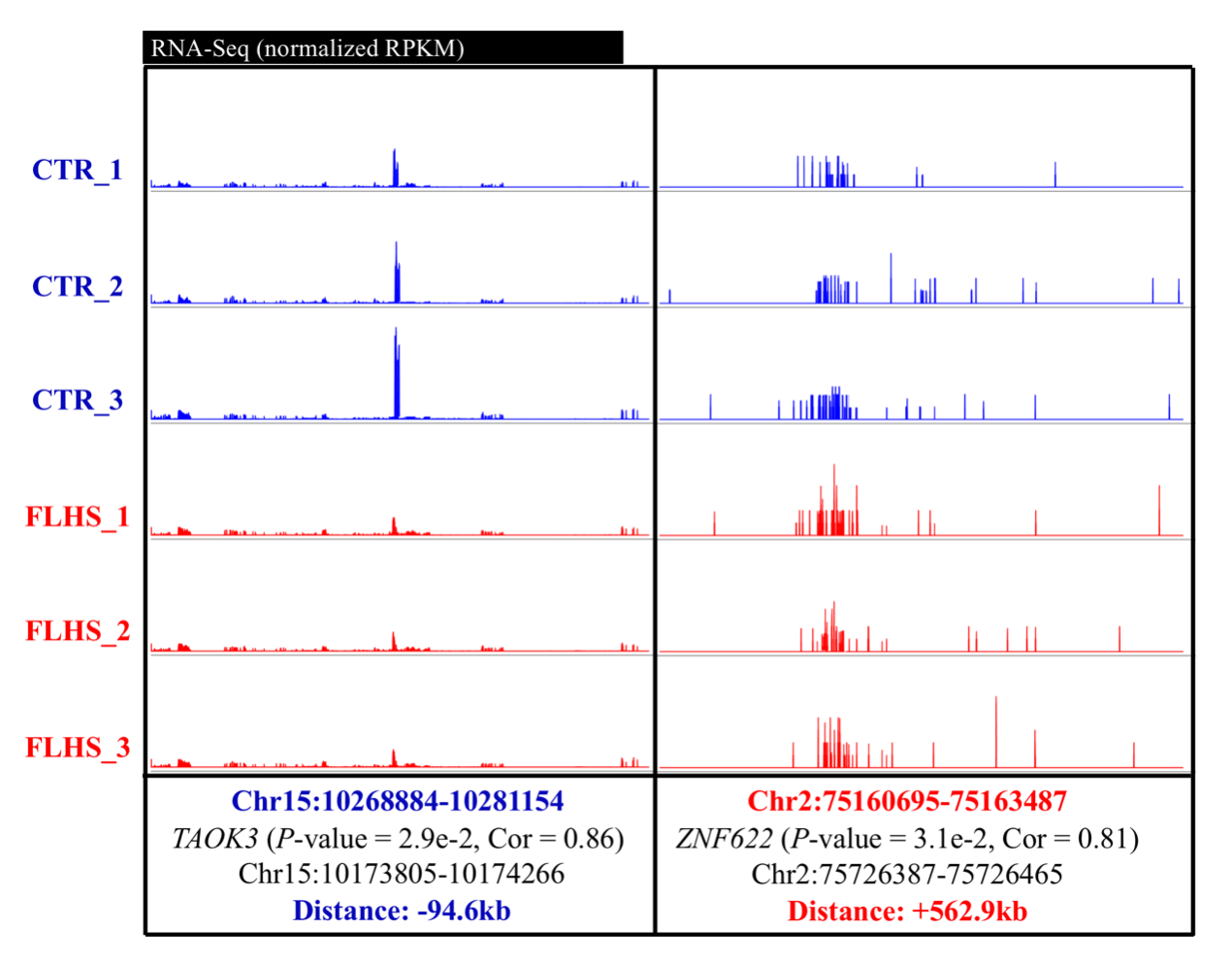
**

**Figure S5. Integrated analysis of H3K27ac CHIP-Seq and RNA-Seq between healthy and FLHS groups.** (A) The number of positive and negative peak-gene interaction in CTR and FLHS. (B) Distribution of peak–gene correlations against peak–gene distance in hypo-acetylated peaks. (C) Distribution of peak–gene correlations against peak–gene distance in FLHS hyper-acetylated peaks. And the frequency in the middle within 100kb were more condense than outside with far distance.

**
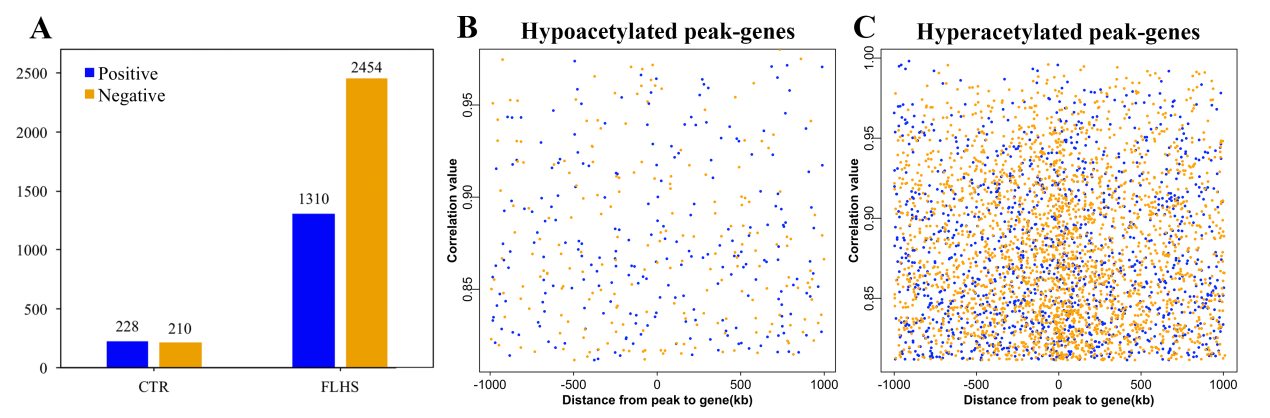
**

**Figure S6. Pathway of cytokine-cytokine receptor interaction.**

**
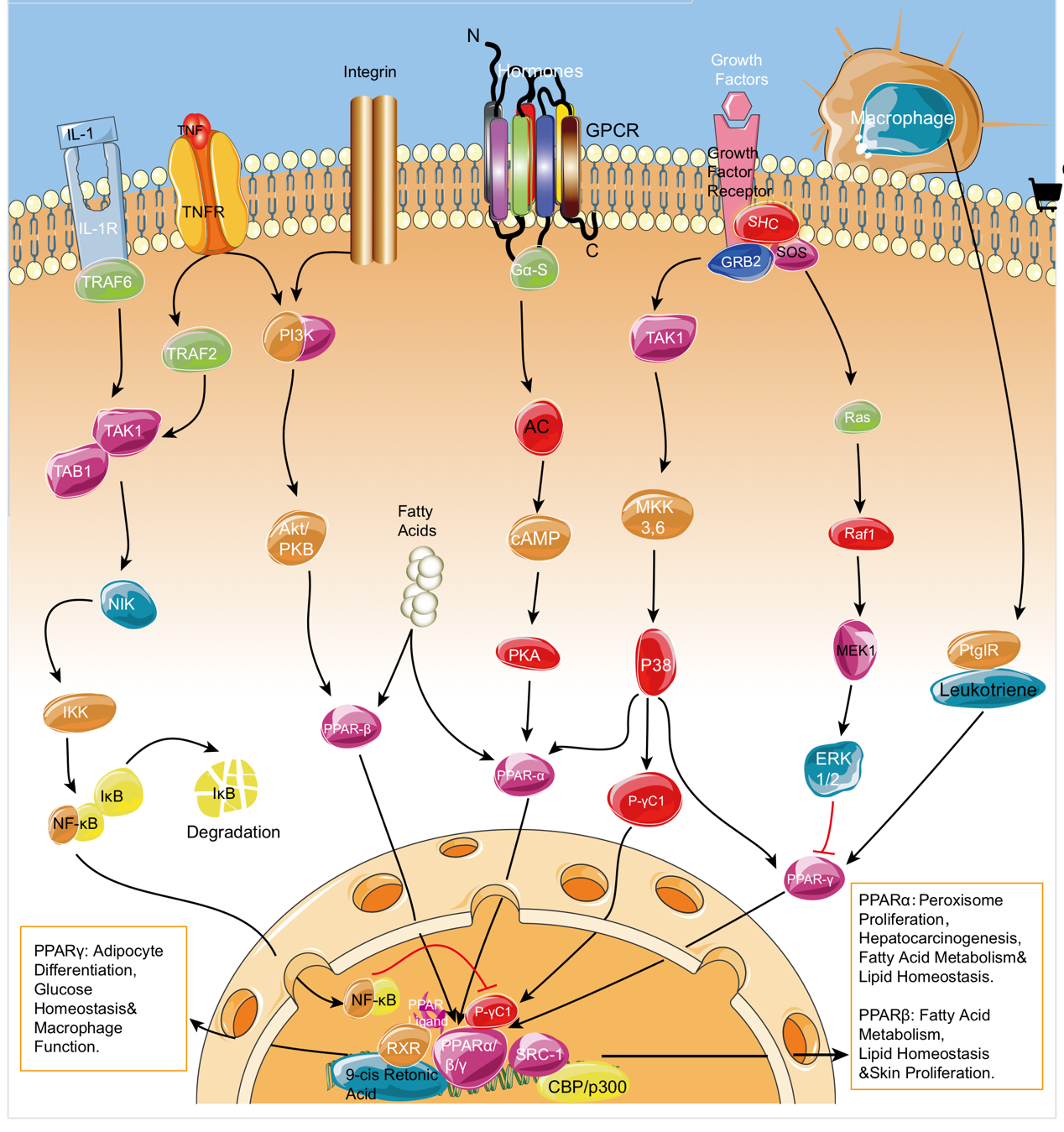
**
